# Asymmetries in Perception of 3D Orientation

**DOI:** 10.1371/journal.pone.0009553

**Published:** 2010-03-04

**Authors:** Allan C. Dobbins, Jon K. Grossmann

**Affiliations:** Department of Biomedical Engineering & Vision Science Research Center, University of Alabama at Birmingham, Birmingham, Alabama, United States of America; University of Minnesota, United States of America

## Abstract

Visual scene interpretation depends on assumptions based on the statistical regularities of the world. People have some preference for seeing ambiguously oriented objects (Necker cubes) as if tilted down or viewed from above. This bias is a near certainty in the first instant (∼1 s) of viewing and declines over the course of many seconds. In addition, we found that there is modulation of perceived orientation that varies with position—for example objects on the left are more likely to be interpreted as viewed from the right. Therefore there is both a viewed-from-above prior and a scene position-dependent modulation of perceived 3-D orientation. These results are consistent with the idea that ambiguously oriented objects are initially assigned an orientation consistent with our experience of an asymmetric world in which objects most probably sit on surfaces below eye level.

## Introduction

**Figure 1:**
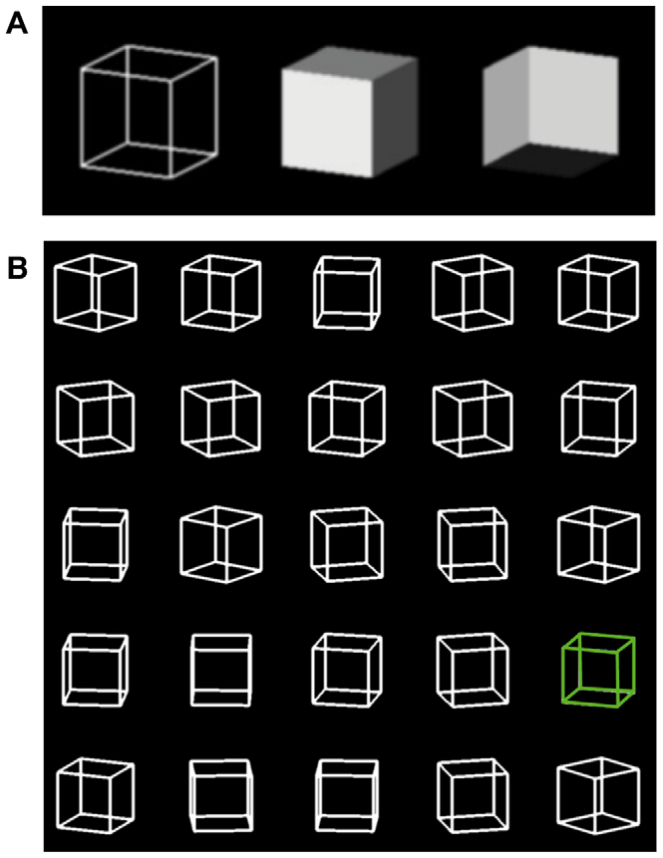
A Necker cube and its two interpretations. **A.** A Necker cube is a 2D projection of a 3D wireframe cube (left). People experience stochastic switching between the two interpretations of orientation shown in the solid cubes. **B.** An array of ambiguous cubes. Observers are very likely to see the cubes as if viewed from above. Rotating the display by 90 degrees eliminates the orientation-based grouping. This is based on one frame of an animation from the first experiment with rotating cubes in which one cube after another is highlighted (green). In the dynamic display (Expt. 1), rotating each cube about a similarly oriented axis links the orientational ambiguity to the rotational ambiguity. For example, a particular cube can appear to be tilted down and rotating to the left, or, tilted up and rotating to the right. Orientation and rotation were randomly coupled throughout the array. If all the cubes were perceived to spin in the same direction, half would appear to be tilted up and half tilted down. Alternatively, if all the cubes were perceived to have the same orientation, half would appear to spin leftward and half rightward. (An animated version of the cube array can be seen at: http://www.vsrc.uab.edu/adobbins.htm).

Sensory information is commonly fragmentary and ambiguous, yet we are compelled to make rapid interpretations and decisions based on the evidence available. When visual scenes are experimentally contrived to contain ambiguities, humans use plausible assumptions or priors to inform the perceptual inference process. These priors reflect the statistical regularities experienced by creatures who orient themselves in a characteristic way in a terrestrial environment. For example light sources are assumed to be above rather than below, an assumption that is widely true in both our natural and engineered environments [1]. Moreover, there is evidence that we favor scene interpretations that hold over a greater range of viewer positions [2] and light source positions [3]. Necker cubes are orthographically projected wire cubes that are inherently ambiguous in orientation (Fig. 1A). It has occasionally been observed that viewers have some preference for seeing a single Necker cube as tilted down rather than up [4]. We have noticed that when people view an array of rotating Necker cubes each with an up/down orientational ambiguity, there is a very strong tendency to see all the cubes as if viewed from above (Fig. 1B). However, when the display is rotated by 90 degrees so that rotation is about a horizontal axis and the orientational ambiguity is left/right, there is no obvious perceptual organization either by orientation or rotation (but see below). In fact one commonly sees an individual cube switch interpretations independently of its neighbors. This asymmetry is evident even in the static display of Figure 1B, in which the cubes are quite stable in one interpretation when viewed with the page upright but metastable when the page is rotated.

## Results

**Figure 2:**
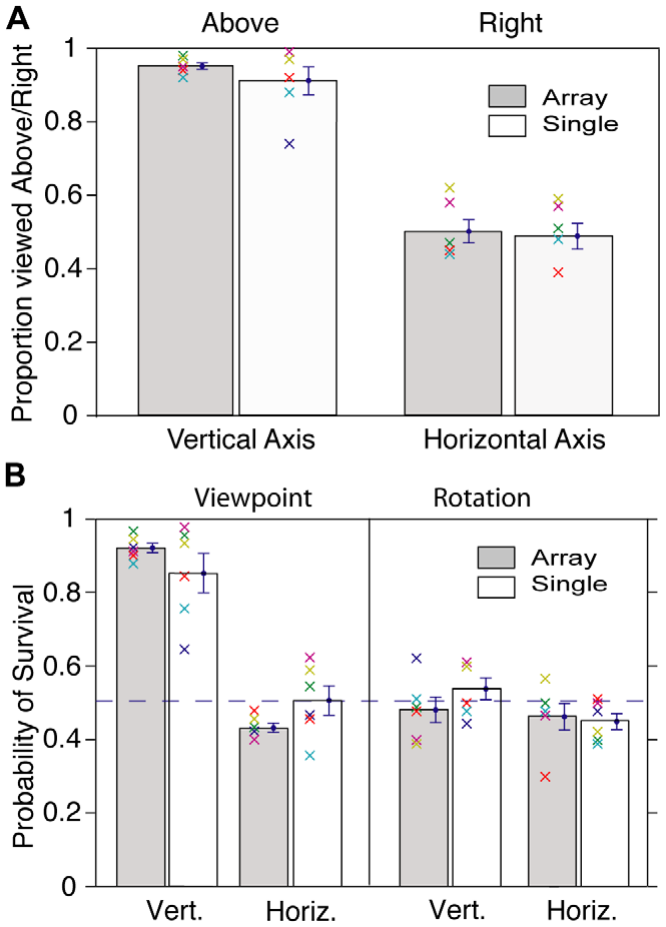
Initial perception bias in a single cube and an array. In a five by five array of cubes, one cube at a time is highlighted (Fig. 1B). In other trials only one cube is present and it appears successively at different positions. **A.** Vertical axis (ambiguous above/below) and horizontal axis (ambiguous left/right). For both the highlighted cube in the array and the single cube there is a profound viewed-from-above bias in the initial percept. However there is no bias in the horizontal axis trials. Colored crosses represent the mean proportion of the individual observers (N = 6). Observe the tighter clustering in the array condition (left column). **B.** Persistence of interpretation from one presentation to the next. The left half of the plot shows orientation/viewpoint persistence and the right half plot shows rotational persistence. Only the tilted up/down interpretation has high persistence. (Mean ± s.e.m.).

We wished to explore the properties of the cube array that serve to amplify or stabilize the orientation bias in Necker cube perception. For instance, it might be the case that the array cooperatively stabilizes one interpretation of the display. To evaluate this hypothesis an experiment was undertaken in which a five by five cube array had one cube after another highlighted with the observer reporting the rotation direction of the highlighted cube. The observer's response triggered the target cube to return to its original appearance and a new cube to be highlighted. Ten cubes were successively highlighted in each trial. In other trials, a single cube appeared at one of the 25 locations of the cube array. In these trials, a keypress caused the cube to disappear and then reappear at a new, random location. The difference between the two trial types is whether an array was visible in addition to the target cube. In this experiment the array was large (22×22 degrees) and observers were free to move their eyes from target to target over the course of each trial. In the vertical axis trials observers reported the target cube as viewed-from-above more than 90% of the time in both array and single cube trials. In contrast, in the horizontal axis trials the individual observers appear to show random scatter about 50% — there was no appreciable bias in favor of either a leftward or rightward viewing position, or for upward or downward rotation (Fig. 2A). The principal difference between the vertical axis array and single cube conditions is the variability among observers: in the array trials all observers showed more than 90% bias, whereas in the single cube trials, two of six observers were below 90%, one substantially so. The array is large enough that one is not aware of the state of all the cubes simultaneously. However the presence of multiple cubes near the target may aid in stabilizing the viewed-from-above bias of the percept, particularly for successive targets that are not too far apart.

Differences in eye movements between observers might also contribute to variability. On the other hand, one would have to postulate different eye movement patterns in the array vs. single cube trials in the same observers. Since the principal difference between the two kinds of trials is whether there are other cubes visible at all times during the trial it seems likely that this factor is the more important.

Moving the eyes from one location to another as one target is replaced by another may have some similarity to interrupted or strobe viewing, which is known to dramatically change the dynamics of perceptual switching (increasing switching rates with rapid gaze shifts (around 100 ms) [5], [6] and slowing switching rates when the interruptions are of the order of a second or two [5], [7]). In vertical axis conditions the first percept is almost invariably the tilted-down interpretation, whereas in the horizontal axis conditions even if the initial percept is arbitrary it might nevertheless persist across the trial. Therefore, one might find no deviation from chance in horizontal axis trials despite high within-trial persistence if there were arbitrary initial choices across trials (N.B. vertical axis trials were always separated by horizontal axis trials). To evaluate this possibility, we analyzed the probability of an interpretation persisting from one target to the next within trials (Fig. 2B). Recall that the orientation/rotation direction coupling varies randomly between successive targets. In the vertical axis conditions, perceived orientation was around 90% likely to persist across targets (array condition) while rotation direction persistence was at chance. Again the inter-subject variability was much smaller in the array condition (compare leftmost two columns). Horizontal axis conditions show no target-to-target persistence for either orientation or spin direction. Therefore it is not the case that there is a within-trial perceptual persistence masked by inter-trial randomness of initial percept in the horizontal axis trials. Hence the asymmetry in horizontal-vertical axis bias cannot be attributed to perceptual stabilization due to interrupted viewing.

**Figure 3:**
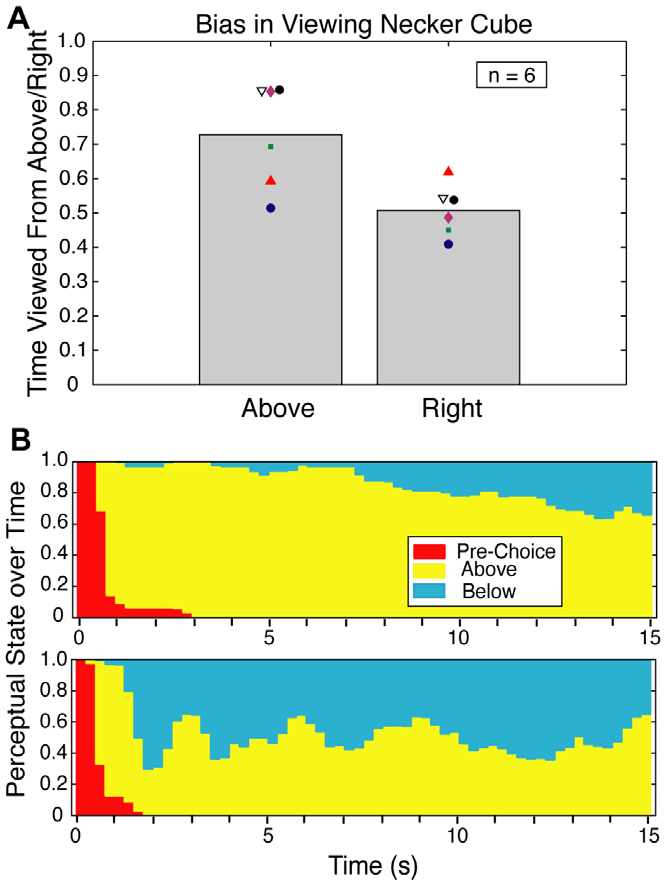
Time evolution of perception for a single rotating cube. **A.** Fraction of time a single rotating cube is seen from above in vertical axis trials (left bar) and from the right in horizontal axis trials (right bar). There is a substantial tilted down/viewed from above bias, but no corresponding bias for tilted left/viewed from right cubes (N = 6). **B.** Peristimulus time histograms for the vertical axis trials for two observers: one of the most biased observers (top) and the least biased observer (bottom). The strongly biased observer shows a gradual decline in the probability of seeing the cube from above over the course of the trial. The neutral observer is as strongly biased as the strongly biased observer over the first second or so of the trial but not thereafter. Pre-Choice (red) represents the time before an initial choice is reported.

Necker cubes are usually described as bistable objects. However in the experiment shown here, when rotating about a vertical axis they are overwhelmingly seen in one orientation. This experiment differs in two respects from the usual situation: the cubes rotate and one looks at a single cube for only a brief moment before moving on to the next one. To evaluate the significance of brief viewing, observers viewed a single rotating cube for 15 seconds and were asked to report each perceptual transition. In the vertical axis trials, on average observers reported seeing the cube tilted down, i.e. from above, about 70% of the time. However, there was significant variation ranging from an observer with no bias to three observers reporting viewed-from-above about 85% of the time. In contrast, in the horizontal axis conditions, while there was individual variation, there was no systematic bias seen across observers (Fig. 3A). Figure 3B shows how perceptual state evolves over time for the most biased and least biased observers in the vertical axis trials. The strongly biased observer (top panel) shows a slow monotonic decline in viewed-from-above probability, suggesting that in a longer trial the average bias would be significantly less. Strikingly, the observer with no average bias (bottom panel) almost invariably first reported seeing the cube as viewed-from-above (consistent with his behavior in Expt. 1). However, due to rapid perceptual switching, the bias disappeared in less than two seconds. This observer demonstrates a complete dissociation between a strong transient initial bias and the long-term average as has been reported previously for binocular rivalry [8]. Together these experiments show that the 3D orientational perceptual bias is greatest in the first instant and declines thereafter.

**Figure 4:**
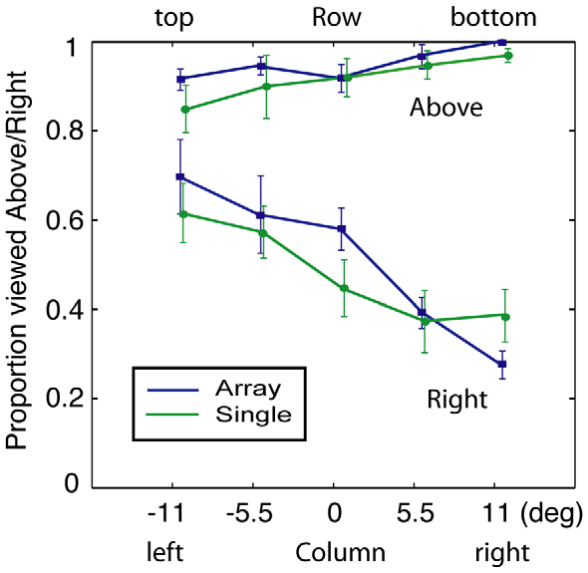
Variation of viewpoint bias with array position. The top two curves show that there is weak modulation of the probability of viewed-from-above with elevation in the array. In contrast, the lower two curves show a very strong modulation of perceived left/right orientation with left/right position – a cube in the leftmost column is much more likely to be seen as if tilted left/viewed from the right. (Mean ± s.e.m.) Least squares linear fits for the horizontal axis conditions: array (y = −1.97 θ+51.37, R^2^ = 0.9672, p = 0.0025) and single cube condition (y = −1.20 θ+47.08, R^2^ = 0.9317, p = 0.0077), where θ is in degrees and y is in percent. Fitting the mean of the paired differences for each observer in the horizontal axis conditions yielded: y = −0.57 θ+3.97, R^2^ = 0.9678, p = 0.0025.

Are object orientation and viewpoint equivalent notions? That is, object orientation depends on the spatial relationship between viewer and object, but one's interpretation of the orientation of a particular object might depend on factors such as gaze angle and scene context. Directing one's gaze upward at an ambiguously oriented object might increase the probability of interpreting it as tilted up or viewed from below. To evaluate this idea, the data from the first experiment were separated by elevation (array row) in the vertical axis conditions, and left-right position (array column) in the horizontal axis conditions. The results, shown in Figure 4, demonstrate a striking modulation of perceived left/right orientation in the horizontal axis trials: objects on the left side of the array are twice as likely to be seen as viewed from the right as objects on the right side of the array (50% more likely for the single cube condition). On the other hand, in vertical axis conditions there is only a weak positional dependence in which objects in the bottom row are more likely to be seen as tilted down (Array. top row: 92%; bottom row: 100%). The much weaker modulation in the vertical axis trials may be a ceiling effect, reflecting the dominance of the viewed-from-above prior in this case. Neurons, beginning in V1 [9]–[11], and in both dorsal [12]–[17] and ventral stream cortical areas [18]–[22] modulate their visual response gain as a function of gaze position. The variation of perceived orientation with gaze angle could reflect a neural gain field effect, but could also be attributable to the direction of approach to the target --- the proximal face being closer to the fovea and therefore enjoy a competitive advantage in representing a visible cube face.

## Discussion

There are many studies demonstrating grouping of ambiguous objects, and others showing that a 3D object that is discrepant in certain ways from its fellows rapidly pops out. For example, arrays of identically oriented equilateral triangles all appear to point in the same direction and switch among the three possible orientations in unison [23]. The exception is when the triangles are oriented base down, in which case they stably appeared to point up, as if sitting on the ground. This latter case is similar to the strong viewed-from-above bias seen in the present experiments. As with Attneave's triangles, Necker cubes joined into complexes along faces or edges may take on interpretations that maximize symmetry of the complex unless a ‘viewed from’ interpretation competes with symmetry [24]. Arrays of bistable dynamic dot quartets appear to have a shared motion axis and switch interpretations synchronously [25], [26]. The presence of a single axis of motion in the visual field would most commonly be caused by an eye or head movement, which may explain the visual system's preference for perceiving a single axis of motion in dynamic dot quartet arrays. When one shaded 3D object in an array differs from the rest in lighting direction or 3D orientation, the discrepant object rapidly pops out [27], [28]. Surface reflectance can also be the basis of rapid visual search [29]. In both sets of studies it was critical that the patterns of brightness in the images be interpreted as 3D objects — similar results were not obtained in planar triptychs — implying that rapid search flags the object that is incompatible with the scene model being generated. However, cast shadows based on an overhead light source can significantly slow search [30]. A recent paper shows that the perceived orientation of a Necker cube depends on its context in an assemblage of a solid, unambiguous cube complex and interpreted the findings in a Bayesian context [31]. The present study shows an asymmetry in a 3D orientation prior that implies that the visual system has encoded the up/down asymmetry of our world: objects tend to sit on the ground or on the desktop below eye level and so are tilted down. This belief is most striking in the first instant of viewing when the perceptual apparatus constructs an interpretation of the image.

Arboreal creatures that rarely descend to earth experience a different visual world. One wonders if the inverted sloth or sometimes topsy-turvy prehensile-tailed primates share our orientational propensities. One might also ask whether humans can learn to overcome their orientational biases in a specific context in which the usual statistics are reversed e.g. in a virtual reality game set on a space station in which the player habitually obtains tools from a tool chest that emerges from the ceiling.

## Materials and Methods

### Ethics Statement

This project was reviewed and approved by the Institutional Review Board for research involving human subjects of the University of Alabama at Birmingham. The review was conducted in accordance with UAB's Assurance of Compliance approved by the Department of Health and Human Services. Written informed consent was obtained from all participants.

### Experimental Setup

Stimuli were created using custom software based on OpenGL and Open Inventor libraries run on a Silicon Graphics Indigo2 and viewed on a 20″ Sony GDM-20 SE2 CRT monitor (video mode: 1280×1024 @ 72 Hz).

### Participants

Both authors and four naïve observers participated in Experiment 1 and one author and five naïve observers participated in Experiment 2.

### Experiment 1

#### Stimulus

A static cube has simultaneous coupled left/right and up/down ambiguities. In contrast, in a rotating cube the orientation ambiguity is coupled to rotation direction. We used rotating cubes because they dissociate up/down from left/right orientational ambiguity and we have found them to be less susceptible to eye [32] and attentional shifts [33] than are static Necker cubes. In addition by asking observers to report rotation direction, orientation is obtained covertly without revealing the purpose of the experiment. Observers viewed a 5×5 array of cubes or a single cube that occupied successive positions. Each cube was 2.5 degrees on a side, pitched 15 degrees out of the plane of the screen (see Fig. 1B), and rotated at 15 rpm with the initial rotational phase chosen randomly for each cube. The cubes were spaced 5.5 degrees apart in both x and y dimensions (total edge-to-edge array size: ∼25×25 degrees). Observers could freely move their gaze and did so.

#### Design

In array trials, one cube was initially highlighted (green) and the observer's task was to report its rotation direction. The response prompted the target cube to revert to white and a new target to be randomly selected. Each trial consisted of ten successive targets. The single cube trials were similar except that the other cubes in the array were not visible. A trial consisted of a cube appearing at one location, a response, cube disappearance and appearance at a new array location, repeated ten times. As in the array trials, cube rotational phase was randomized. Because of the large array size, observers moved their eyes from target to target over the course of a trial. Each block of four trials consisted of array and single cube trials for both horizontal and vertical axis. Experimental sessions consisted of ten blocks of trials.

### Experiment 2

#### Stimulus

A single orthographically projected, wireframe cube rotated either about a horizontal or a vertical axis in a 15 second trial. The cube was comprised of white line segments on a black background. The cube was 3 degrees on a side and rotated at 20 rpm. The cube and rotational axis were tilted 12 degrees out of the plane of the monitor.

#### Design

Prior to the beginning of a trial, the observer was cued to use either the Left and Right arrow keys (vertical axis trials) or the Up and Down arrow keys (horizontal axis trials). A first bar press initiated the fixation spot (red, 0.2 degrees) and a second bar press caused the cube to appear. The fixation spot and cube were centered on the screen.

The head was restrained by a forehead/chin rest 57 cm from the display adjusted so that the eye height was centered. Observers were instructed to maintain fixation throughout the trial and promptly indicate each perceptual transition. Cubes rotated left/right or up/down in 15 second trials. Orientation, rotation direction, and rotational axis were randomized creating four trial types. Successive trials alternated between vertical and horizontal axis rotation. A session consisted of 16 four trial blocks for a total of 16 minutes of viewing time. The day before the experiment each observer participated in a practice session identical to the experiment to develop fluency in the task and to eliminate the tendency to see only one interpretation when first viewing bistable stimuli.
